# Dose-Response Behavior of Dental Material Using General Kinetic Order and Lambert W Deconvolution Models in CW-OSL

**DOI:** 10.3390/mps8050112

**Published:** 2025-10-01

**Authors:** Ioanna K. Sfampa

**Affiliations:** Nuclear and Elementary Physics Laboratory, School of Physics, Faculty of Sciences, Aristotle University of Thessaloniki, GR-54124 Thessaloniki, Greece; isfaba@physics.auth.gr

**Keywords:** ZLS, OSL, Lambert W, GOK, dose-response, deconvolution, accidental dosimetry

## Abstract

The present study presents a comparative evaluation of two analytical deconvolution models applied to Optically Stimulated Luminescence (OSL) decay curves of zirconia-reinforced lithium silicate (ZLS), a glass-ceramic material with potential applications in accidental dosimetry. ZLS samples were subjected to beta irradiation and measured under Continuous Wave OSL (CW-OSL) protocols. A comparative analysis is conducted between two deconvolution approaches—the General Order Kinetics (GOK) model and a master analytical equation based on the Lambert W function. The results imply that both models yield a linear dose-response behavior of the fast OSL component; however, the Lambert W approach offers simpler fitting with fewer parameters. The abovementioned findings demonstrate the methodological robustness of the Lambert W formalism and also confirm that ZLS is a promising dosimetric material, aligning with the goals of protocol development in material characterization.

## 1. Introduction

Over the years there have been significant advancements in radiation safety protocols. Nevertheless, public concern remains high regarding the potential consequences of radiation incidents, ranging from nuclear accidents and radiation leaks to the overexposure of patients during medical treatments or technical staff during laboratory procedures [1,2]. The abovementioned scenarios have intensified the demand for Accidental Retrospective Dosimetry, which aims to determine accurately the absorbed doses using post-event measurements [3,4]. Luminescence techniques, such as Optically Stimulated Luminescence (OSL), are widely recognized as powerful tools in this direction due to their sensitivity, reproducibility, and compatibility with a broad variety of natural and synthetic materials [5,6,7]. Among potential candidates, dental restorative materials present a unique advantage as they are attached to individuals and their sampling involves minimal discomfort, at least compared to biological tissues. Consequently, materials such as feldspathic porcelains, alumina-based ceramics, zirconia composites, and glass-ceramics have drawn increasing attention for accidental dosimetry studies [8,9,10,11,12,13,14]. It is important to mention that OSL materials exhibit an extremely high dynamic range (eight orders of magnitude), reflecting the relationship between the luminescent signal and the dose [15]. Equally important to the material response are the reliability and speed of data analysis; accurate and efficient curve deconvolution methods are critical for extracting meaningful dose-response information and providing fast, safe conclusions during emergency scenarios.

Specifically, OSL has been proven as a valuable tool in the fields of radiation dosimetry and material characterization due to its sensitivity, stability, and the variety of analytical models available for interpreting its decay behavior [16,17]. One of the key aspects in OSL data analysis is the computerized curve deconvolution (CCD) of the luminescence decay curves into their constituent components. This process provides critical insight into the trapping and recombination processes involved, and it enables a quantification of the radiation doses absorbed by materials.

Over the past decades, a wide range of deconvolution methodologies has been developed based on fundamental kinetic models. Among them, the General Order Kinetics (GOK) model has been widely adopted due to its versatility in capturing first-, second-, and intermediate-order processes [18]. These classical models have proven effective but often require multiple components and careful parameterization.

In parallel, a few years ago Kitis & Vlachos introduced an alternative analytical framework based on the Lambert W function [19], which is derived from the One Trap One Recombination (OTOR) model [20,21,22,23,24]. This approach has led to master equations that describe several types of luminescence stimulation—such as Thermoluminescence (TL) and Continuous Wave OSL (CW-OSL) or Linearly Modulated OSL (LM-OSL)—under a common formalism [25,26]. These equations, once expressed in terms of experimentally accessible fitting parameters, allow direct fitting of experimental data with fewer free parameters. This approach not only simplifies the analysis but also retains strong physical interpretability [27].

Zirconia-reinforced lithium silicate (ZLS), a glass-ceramic material widely used in dental applications, has recently emerged as a potential candidate for passive personal dosimetry [28]. Preliminary work by Sfampa et al. has demonstrated that ZLS exhibits a dominant TL peak at ~190 °C, stable and reproducible glow curves over repeated cycles, linear dose–response in the TL signal, a lower detectable dose limit of ~30 mGy, and negligible fading under dim conditions, with an activation energy of ~1.01 eV for the main trap [29]. These results confirmed its reproducibility, stability, and dose linearity for the case of TL, highlighting its suitability for luminescence-based dosimetry. However, the behavior of this material, particularly in OSL mode, and the robustness of different deconvolution strategies remain largely unexplored.

This work aims to investigate the OSL dose-response characteristics of ZLS samples using two distinct analysis approaches: the traditional GOK model and the Lambert W-based master equation. Particular emphasis is placed on the linearity of the dose-response, the kinetic order of the fast OSL component, and the practical advantages of each model in terms of fitting accuracy and simplicity. The results contribute to the understanding of ZLS as a dosimetric material and offer insights into the optimal deconvolution methodology for analyzing complex luminescence signals. Such comparative evaluations are essential for developing reliable and transferable analytical protocols, especially for interdisciplinary applications in accidental dosimetry.

The novelty of this work lies in the combined application of the GOK and Lambert W analytical approaches to zirconia-reinforced lithium silicate, a material that has not yet been thoroughly characterized for its OSL behavior. Understanding its dose-response properties is critical for the development of a new personal dosimetry system, especially for scenarios of accidental or emergency exposure where quick and reliable dose assessment is needed. By evaluating both the material and the methodology in a single comparative study, this work bridges the gap between fundamental luminescence modeling and practical protocol optimization. The presented results not only provide direct evidence of ZLS’s linear response to radiation but also present a simplified deconvolution method through the Lambert W formalism, possibly influencing future analytical frameworks in dosimetry and materials science.

## 2. Materials and Methods

### 2.1. Sample Preparation

The studied specimens were fabricated from a zirconia-reinforced lithium silicate (ZLS) glass-ceramic, commercially known as VITA SUPRINITY^®^ (VITA Zahnfabrik, Bad Säckingen, Germany) [29]. Rectangular ZLS specimens were cut at dimensions 10 × 10 × 1.5 mm using a high-speed rotary handpiece and diamond discs under water cooling. The specimens were cleaned with distilled water in an ultrasonic bath (15 min), dried, and left as received without additional polishing. All aliquots were fabricated from the same commercial block to ensure compositional and microstructural uniformity.

All specimens were cut into suitable aliquots for luminescence experiments.

### 2.2. Instrumentation and Dosimetric Protocol

The luminescence measurements were performed using a Risø TL/OSL reader (model TL/OSL DA-15), equipped with a calibrated Sr^90^/Y^90^ beta radiation source delivering a dose rate of 0.0521 Gy/s. The irradiator is made of brass and is surrounded by lead. Furthermore, an aluminum safety helmet covers the entire irradiator and lead shielding. The source is mounted into a rotating, stainless steel wheel, which is pneumatically activated (using nitrogen flow). The distance between the source and the sample is 5 mm. For the OSL measurements, six clusters of seven blue LEDs (λ = 470 ± 20 nm) were used, delivering a total irradiance of ~40 mW cm^−2^. At 82% of maximum power, the irradiance was ~33 mW cm^−2^. The LEDs were arranged in a ring-shaped holder at ~25 mm from the sample. Luminescence was detected through a 7.5 mm Hoya U-340 filter by an EMI 9235QA bialkali PMT with extended UV sensitivity (200–400 nm). All measurements were conducted under a constant nitrogen atmosphere to suppress oxidation and minimize background luminescence.

The experimental protocol included the following:

- Step 1: Zeroing of Signal: All samples were thermally pre-treated by heating up to 773 K to erase any residual signal.
- Step 2: Irradiation: Samples were exposed to different beta doses (namely, D_i_ = 0.5, 1, 2, 4, 8, 16, 32, 64, 128 Gy).
- Step 3: Thermal Cleaning: TL readout up to 393 K at constant increase of 2 K/s was used to remove shallow traps.
- Step 4: CW-OSL Acquisition: CW-OSL was measured at room temperature for 250 s using 82% of the maximum stimulation intensity.
- Step 5: Post-OSL TL: Final TL glow curve was recorded up to 773 K at 2 K/s to monitor residual charge (residual TL—RTL).

Previous work on the same material [29] has shown that repeated TL cycles up to similar temperatures do not induce sensitivity loss or signal drift, and no phase or color changes were observed, confirming the stability of ZLS. Based on this evidence, the present work applies a single-aliquot protocol. To ensure reproducibility, each experimental cycle was repeated on three independent aliquots, and the mean value was used for all dose-response calculations. The relative standard deviation of repeated measurements was consistently below 3%, confirming the stability of the experimental protocol. Calibration of the Sr^90^/Y^90^ beta source was verified prior to the experiments using a reference dosimeter, ensuring that the delivered dose rate remained within ±2% of the nominal value.

Additional checks were performed to quantify potential sources of uncertainty, including fluctuations in stimulation intensity and background signal variability. These uncertainties were combined using a standard propagation of errors approach, leading to an overall estimated uncertainty of less than 5% for all integrated CW-OSL signals. Such an error assessment is important for establishing the reliability of the derived dose-response curves.

### 2.3. Curve Deconvolution Approaches

The OSL decay curves were analyzed using two independent fitting approaches. All fittings were performed using customized fitting routines and Microsoft Excel Solver [30,31], optimized for least-squares convergence between experimental and theoretical curves and changing, accordingly, the used equations.

#### 2.3.1. General Order Kinetics (GOK) Model

OSL curves were first deconvoluted using the General Order Kinetics (GOK) formalism [18,19], which enables resolution into individual components with kinetic orders ranging between first and second. For CW-OSL, the decay curve was transformed into a pseudo-Linearly Modulated OSL (pseudo-LM-OSL) format, as follows:

(1) $$u=\sqrt{2tP_{cw}}$$

where u represents the pseudo-time, t corresponds to real time, and P_CW_ is the total duration of the CW-OSL stimulation. Using this transformation the featureless CW-OSL decay I(t) is transformed into the following pseudo-LM-OSL intensity:

(2) $$I\left(u\right)=u\frac{I(t)}{P_{CW}}$$

Finally, the used equation is

(3) $$I\left(u\right)=I_{m}\frac{u}{u_{m}}{\left(\frac{b-1}{2b}\frac{u^{2}}{u_{m}^{2}}+\frac{b+1}{2b}\right)}^{\frac{b}{1-b}}$$

where I_m_ is the maximum intensity, u_m_ is the value of the pseudo-time at the maximum of the curve, and b represents the kinetic order (1 ≤ b ≤ 2).

#### 2.3.2. Lambert W-Based Master Analytical Equation

In parallel, the experimental CW-OSL data were fitted using the analytical solution of the OTOR model, based on the Lambert W function [19,20,26].

The original equation derived by [1] as a solution of the OTOR model is

(4) $$I\left(t\right)=\frac{N\;R}{{\left(1-R\right)}^{2}}\frac{p(t,T)}{W\left(z\right)+W{\left(z\right)}^{2}}$$

where N is the initial trap occupancy, R is the retrapping ratio, and W(z) is the Lambert W function. In the general form of the OTOR model, the stimulation is described by the function p(t,T), which may depend both on time and temperature. Specifically for CW-OSL, performed at room temperature, the stimulation is expressed by the function p(t) = λ, where λ (s^−1^) is the decay constant of the model related to the mean lifetime by τ = 1/λ. So, the parameter z of the Lambert function W(z) becomes equals to

(5) $$z=exp\left(\frac{R}{1-R}-ln\left(\frac{1-R}{R}\right)+\frac{\lambda t}{1-R}\right)$$

Here, R denotes the retrapping ratio, a dimensionless parameter indicating the kinetic order, with R ≪ 1 corresponding to 1st-order kinetics and R → 1 to 2nd-order. This approach enables direct fitting of raw experimental data with fewer adjustable parameters, offering practical advantages for dosimetric applications.

## 3. Results

**Figure 1:**
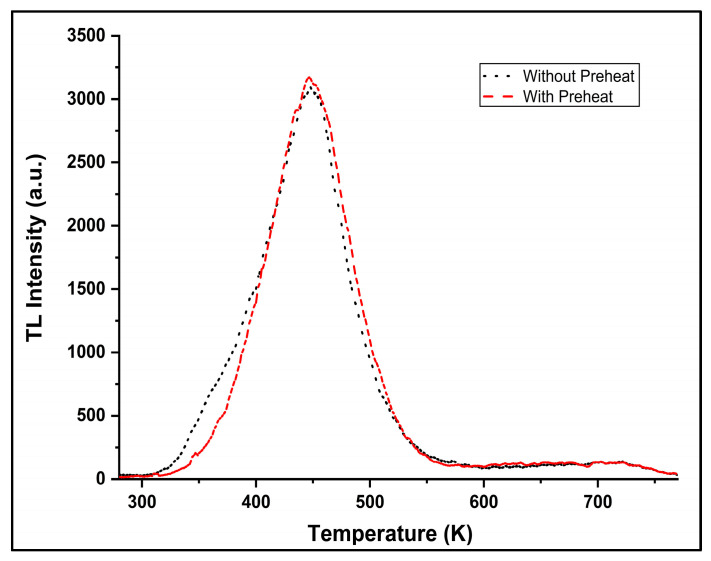
Thermoluminescence (TL) glow curves of ZLS after beta irradiation, recorded with preheat at 393 K (red) and without preheat (black).

First of all, the effectiveness of Step 3 of the protocol, which aims at emptying the shallow traps, is demonstrated in Figure 1. As can be seen, the low temperature side of the glow curve is effectively reduced. This fact indicates the successful removal of shallow traps. This result confirms that the fast OSL component analyzed in the following steps is not affected by residual signals from shallow traps. Preheats of this order are widely employed in OSL protocols [32,33] and are well documented not to deplete the fast OSL trap but only to remove unstable shallow traps.

The OSL decay curves were successfully recorded following irradiation at varying beta doses. Deconvolution of the CW-OSL curves revealed distinct components corresponding to different trap populations. The primary focus was on the first component, which is usually referred to as a fast component since it corresponds to a trap with a short lifetime. This component is of particular relevance for dosimetric applications due to its prompt and intense signal.

### 3.1. Comparison of Fitting Methods: GOK vs. Lambert W

**Figure 2:**
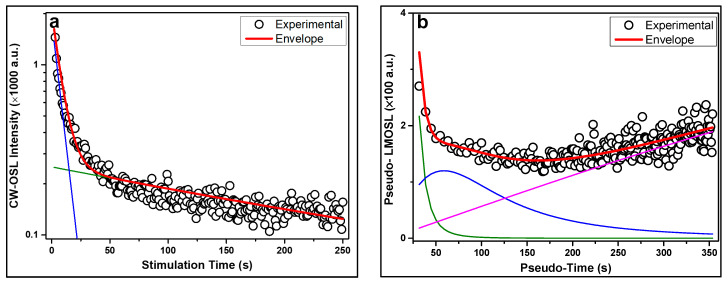
Representative CW-OSL decay curve fitting using two different deconvolution models: (**a**) fitting with Lambert W-based master analytical equation; (**b**) fitting with GOK equations. Experimental data are shown as black circles, while the envelope of the theoretical fit (sum of all the components) is represented by the thick red line. The remaining colored lines (blue, green, and magenta) correspond to the individual components.

Figure 2 illustrates a comparative deconvolution of a representative CW-OSL decay curve using two analytical approaches examined in this study. In Figure 2a, the fitting was performed using the Lambert W formalism. The model reproduces the experimental signal with high accuracy by using only two components, suggesting a more compact and physically grounded description of the underlying trap population. The fast component dominates the early part of the decay curve, while a slower one accounts for the residual signal at longer stimulation times.

On the other hand, the right side of the figure (Figure 2b) presents the GOK model fit, where the CW-OSL data were first transformed into a pseudo-LM-OSL curve. In this case three components were required in order to achieve an equally good fit, as shown by the colored sub-curves and the overall envelope. Although the GOK model offers flexibility in kinetic order, the need for more parameters introduces potential over-fitting and reduces the interpretability of the individual components.

Nevertheless, in both approaches, the first component, the dominant one, consistently exhibited characteristics of first-order kinetics, reinforcing its relevance for dosimetric applications due to its linear dose-response and strong signal intensity. However, the Lambert W-based approach offers notable advantages in terms of fitting simplicity and parameter stability, which is crucial for systematic analysis. This reduction in the number of required components minimizes the risk of overparameterization compared to the GOK approach.

### 3.2. Dose-Response Behavior

**Figure 3:**
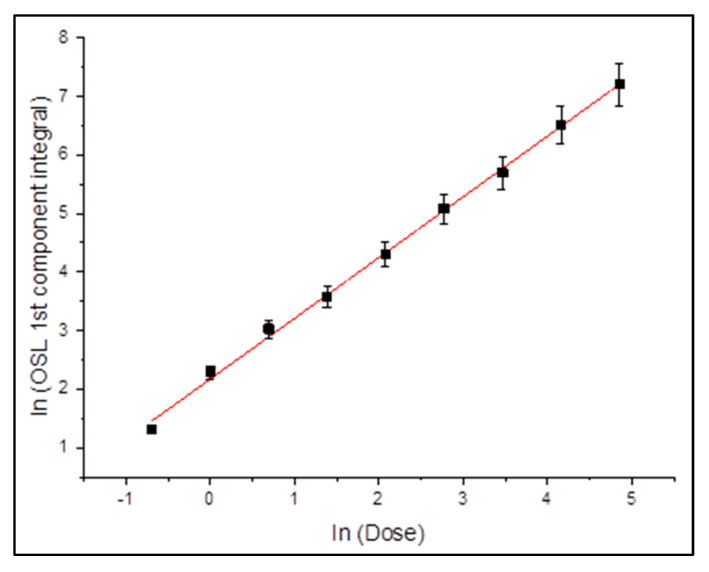
CW-OSL dose response for the 1st component for the case of fitting with GOK equations. Black squares represent experimental data, while the red dashed line denotes the linear regression fit. Error bars are smaller than 3% for all data points.

**Figure 4:**
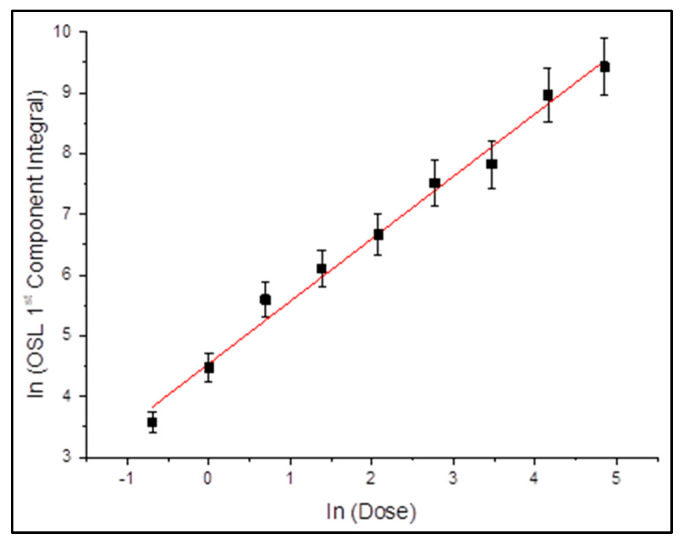
CW-OSL dose response of sample A for the 1st component for the case of fitting with Lambert W-based master analytical equation. Black squares represent experimental data, while the red dashed line denotes the linear regression fit. Error bars are smaller than 5% for all data points.

The dose response of the fast component was extracted for each fitting model. Figure 3 and Figure 4 present the integrated signal (log-scale) as a function of the logarithm of the delivered dose.

Concerning Figure 3, which presents the dose-response behavior of the first (fast) component extracted by using the GOK model, the data are displayed on a logarithmic scale for both axes. The logarithmic scale facilitates linearity evaluation across a wide range of doses, namely 0.5–128 Gy. The linear regression yields a slope of 0.98 ± 0.03 and a coefficient of determination R^2^ = 0.997, signifying that the fast component exhibits a highly linear response to the absorbed dose.

This result confirms that the fast component follows an almost ideal linear dose response, validating its reliability for dosimetric purposes. The low dispersion of data points and minimal residuals confirm that the GOK fitting provides a statistically sound description of the signal. Moreover, the fact that the fast component alone captures the trend without requiring high-order corrections underlines its physical significance in the trapping-recombination processes involved in ZLS ceramics.

Figure 4 illustrates the dose-response behavior of the fast CW-OSL component as derived from the fittings with the Lambert W-based equation. The data, plotted in logarithmic axes, present an almost ideal linear trend with a slope of approximately 1.1 and a coefficient of determination, R^2^, being almost equal to 0.996. The distribution of data points closely follows the regression line, indicating that the Lambert W model accurately captures the underlying signal behavior across the entire dose range. No significant deviations or systematic errors are observed in the residuals, reinforcing the physical consistency of the model. These results are in good agreement with those obtained from the GOK fitting (Figure 3), further supporting the fact that the fast component is a reliable indicator for absorbed dose. It is worth mentioning that the Lambert W model achieves this with fewer fitting components, strengthening its computational efficiency and suitability for practical dosimetric protocols.

Both fitting methods yielded a linear dose-response relationship over the studied dose range (0.5–128 Gy). The slopes obtained from linear regression were close to unity. This consistency confirms the predictable behavior of the fast CW-OSL component with respect to absorbed dose, a fundamental requirement for reliable dosimetric performance. The convergence of the results, both from GOK and Lambert W, further strengthens this trend and supports the suitability of ZLS as a candidate material for personal accidental dosimetry.

## 4. Discussion

The present comparative application of two different analytical deconvolution models—namely, the GOK and the Lambert W-based master equation—offers valuable insight into both material behavior and methodological suitability. Within the context of this study, the analytical aim focuses not only on the performance of ZLS under irradiation but also on the ability of each model to strongly and efficiently interpret the CW-OSL signal.

The observed linear dose-response is a key requirement for dosimetric materials, ensuring predictable and proportional signal output with increasing dose. Both deconvolution approaches, GOK and the Lambert W-based analytical model, yielded similar linearity and slope values for the fast component. This agreement validates the physical strength of the observed trend and confirms the presence of well-behaved, first-order trap dynamics, as also indicated by the low retrapping probability (R ≪ 1) obtained from the Lambert W fits [19,20]. Such linearity reflects the behavior previously reported in thermoluminescence (TL) studies of the same material [29], reinforcing the reproducibility of the ZLS luminescence mechanisms across different stimulation modes.

The parameter R, which is extracted from the Lambert W model analysis, presents an almost stable behavior across the dose range of 0.5 to 64 Gy, whereas values slightly differ between 0.0058 and 0.0067. These values indicate that the first fast component follows first-order kinetics, with minimal retrapping influence. However, at a higher dose of 128 Gy, a significant increase in R is observed, reaching the value of 0.44. This sharp change suggests a deviation from first-order kinetics, potentially due to the progressive filling of traps and the subsequent activation of retrapping pathways at high dose levels. The required exposure time for this dose was ~41 min at the given dose. During these irradiations, no evidence of sample heating, color change, or phase alteration was observed. For lower doses (0.5–64 Gy), exposure times ranged from ~10 s to ~20 min with equally stable behavior. Possible instrumental effects such as PMT nonlinearity or dead-time were considered; however, the EMI 9235QA PMT used in the Risø DA-15 reader is designed for TL/OSL applications and operates linearly under the photon flux levels of the present measurements. No signs of saturation or distortion were observed in the raw decay curves. Therefore, the deviations are attributed to intrinsic dose-dependent changes in recombination probability and trap stability rather than detector artifacts.

This behavior aligns with observations in other well-established dosimetric materials such as BeO, where fitting using Lambert W-based analytical expressions—such as those proposed by Konstantinidis et al. [20]—has enabled accurate curve deconvolution and dose-response analysis in a reproducible and computationally efficient manner. The analysis in the abovementioned study showed that the model successfully captures first-order kinetics at low to moderate doses and highlights deviations at higher dose levels, attributed to the activation of retrapping or complex recombination mechanisms. This consistency supports the reliability of the Lambert W formalism in describing dose-dependent kinetic behavior across different material systems.

Furthermore, concerning the lifetime parameter (*τ*) derived from the Lambert W analysis, a noteworthy trend is revealed. For lower doses—from 0.5 Gy up to 64 Gy—*τ* remains almost stable to high values—typically close to or above 10^5^ s—indicating the presence of a dominant, deep, and stable trapping center. This behavior implies a strong resistance to trap saturation and suggests that, within this dose range, the fast component is mainly a single, well-isolated trap-recombination system, likely associated with intrinsic defects in the lithium-silicate matrix stabilized by zirconia inclusions. However, at the highest dose of this study (128 Gy), a sharp reduction in τ is observed, dropping below 500 s. This sudden transition may indicate the beginning of alternative recombination channels, activation of shallower traps, or saturation of the dominant trap due to structural modifications or local heating effects under intense irradiation.

Similar trends have been also reported in the literature. Konstantinidis et al. [20] demonstrated that in materials such as BeO, the *τ* parameter remains stable at low to intermediate doses, but exhibits marked reductions at higher doses (>30 Gy), correlating with increased retrapping and the emergence of complex recombination pathways. Kitis & Vlachos [19], using the same Lambert W equations, highlighted that changes in τ and R under heavy irradiation reflect kinetic transitions from first-order to quasi-second-order behavior, indicative of trap competition and carrier redistribution. Similarly, Sholom & McKeever [17] emphasized the importance of tracking such kinetic changes in emergency dosimetry protocols, as luminescent materials may display non-linear responses at elevated dose levels, putting at risk the accuracy of dose estimation. The convergence between our findings in ZLS and those reported in other well-established dosimetric materials reinforces the general validity of τ as a sensitive kinetic indicator and further confirms the suitability of the Lambert W approach for identifying such transitions.

From a methodological standpoint, the ability to reduce fitting complexity while maintaining quantitative accuracy is an important advantage in protocol development, especially for applications requiring reproducibility, automation, or high-throughput analysis. The Lambert W model aligns well with these requirements and offers a broader use in luminescence-based characterization protocols.

In the broader context of analytical methods in retrospective dosimetry, so indirectly in natural sciences and archaeometry, this study demonstrates how model selection and protocol design directly influence the quality and interpretability of luminescence data. Such dual evaluations—examining both material and method—are essential for advancing the standardization and cross-disciplinary applicability of luminescence techniques in dating, dosimetry, and materials science.

Finally, the Lambert W-based approach, due to its efficiency and physical grounding, presents a compelling option for future analytical protocols where simplified yet reliable modeling of OSL data is required.

## 5. Conclusions

This study presents, for the first time, a comprehensive evaluation of the CW-OSL dose-response of zirconia-reinforced lithium silicate (ZLS) ceramics, using both the General Order Kinetics (GOK) model and a master analytical equation based on the Lambert W function.

The main findings are as follows:

- The dose-response of the fast OSL component was found to be highly linear across a wide dose range; specifically, it was tested between 0.5 and 128 Gy, regardless of the fitting model used.
- The Lambert W-based deconvolution proved to be more efficient, since through this procedure fewer components were required, and it offered a physically grounded framework suitable for direct use with CW-OSL data.
- Across the 0.5–64 Gy dose range, the retrapping ratio (R) remained low and stable (0.0058–0.0067), suggesting consistent first-order kinetics and confirming the reliability of the fast component as a dosimetric signal. At 128 Gy, R increased sharply to 0.44, indicating a shift to more complex recombination dynamics.
- The lifetime parameter (τ) was consistently high (≈10^5^ s) across the same range, denoting stable traps. Its sharp reduction at 128 Gy suggests the activation of secondary traps or changes in recombination behavior under heavy irradiation.

These results collectively support the potential of ZLS as a reliable candidate for personal accidental dosimetry, combining favorable luminescence characteristics with long-term material stability. Specifically, at 128 Gy, both the sharp increase in R and the reduction in τ suggest that the dominant trap begins to saturate, and recombination dynamics become more complex. Such behavior has been attributed in the literature to the progressive filling of deep traps and the involvement of secondary, shallower traps or recombination centers, leading to deviations from first-order kinetics. Furthermore, this study confirms the applicability of the Lambert W master equation as a practical and strong tool for luminescence signal analysis in advanced dosimetric materials for the following reasons:

- It requires fewer fitting components, reducing the risk of overparameterization.
- It is directly based on the physical OTOR model, yielding parameters with clearer physical meaning.
- It allows fitting of raw CW-OSL data, without the need to transform into a pseudo-LM format.

These features make the Lambert W approach particularly attractive for routine or automated analysis of CW-OSL data in dosimetric systems, where speed and accuracy are critical. Nonetheless, the GOK model remains valuable for comparative purposes and for studying complex systems with multiple overlapping trapping centers.

Moreover, the demonstrated agreement between the classical and the semi-analytical models underscores the reliability of the proposed protocol, not only for academic modeling but also for real-time dose estimation scenarios. Quantitative agreement was assessed through regression slopes, coefficients of determination (R^2^), and residual analysis. The similarity of these metrics confirms that both models reliably describe the fast OSL component, reinforcing the robustness of the observed linear dose-response. The reduced complexity of the Lambert W approach offers a clear advantage for implementation in embedded software or portable reader systems, potentially enabling field transportable dosimetric solutions using common materials, even attached to humans, such as ZLS of dental restorations.

The conclusions may be summarized in the following table. A direct comparison of key characteristics between the two deconvolution models is presented in Table 1, highlighting the advantages of the Lambert W model in terms of simplicity and automation potential.

It should be noted that since the Lampert W approach is directly linked to the OTOR model, its applicability is naturally limited to materials that do not exhibit multiple trapping centers and complex recombination pathways. Nevertheless, in the case of ZLS, the results indicate that the use of this formalism is a meaningful and efficient way of describing the material signal.

While the present study provides a strong comparative evaluation of two analytical deconvolution models for ZLS ceramics, several limitations remain. The current analysis focuses primarily on beta irradiation, without assessing the sensitivity to other radiation types such as X-rays or heavy charged particles, or studying the behavior for doses above 128 Gy. In addition, long-term fading tests were beyond the scope of this work but are essential for validating ZLS as a dosimetric material in real-world conditions.

Future work should also investigate the influence of material aging and mechanical stress (e.g., grinding or thermal cycling) on OSL properties. Given that ZLS is already used in dental restorations, it offers an inherent advantage as a passive dosimeter attached to individuals. Calibration of its OSL response and development of non-destructive readout protocols could enable practical integration in accidental dosimetry systems. However, issues such as compositional consistency across batches and the influence of dental processing steps must be addressed in future work. Incorporating machine-learning-based curve fitting could further enhance the robustness and automation of deconvolution, particularly when analyzing large datasets, since low parameter count, direct fitting, and closed-form expressions make Lambert W-based analysis particularly suited for automated or real-time OSL analysis in portable dosimetric devices. Finally, exploring co-doping strategies to tune trap depths and improve sensitivity may open new opportunities for customized glass-ceramic dosimeters.

## Figures and Tables

**Table 1:** Comparative features of the GOK and Lambert W models for CW-OSL signal deconvolution.

Feature	GOK	Lambert W
Components required (no.)	3	2
Fitting complexity	High	Lower
Physical basis	Empirical (generalized)	OTOR analytical solution
Interpretability of parameters	Medium (model dependent)	High (physically meaningful R, τ, λ)
Need for data transformation	Yes	No
Automation potential	Moderate	High

## Data Availability

The raw data that support the findings of this study are available from the corresponding author upon reasonable request.

## Abbreviations

The following abbreviations are used in this manuscript:

OSL	Optically Stimulated Luminescence
ZLS	Zirconia-reinforced Lithium Silicate
CW-OSL	Continuous Wave Optically Stimulated Luminescence
GOK	General Order Kinetics
LM-OSL	Linearly Modulated Optically Stimulated Luminescence
CCD	Computerized Curve Deconvolution
OTOR	One Trap One Recombination
TL	Thermoluminescence
RTL	Residual Thermoluminescence
